# Application of Tryptophan and Methionine in Broccoli Seedlings Enhances Formation of Anticancer Compounds Sulforaphane and Indole-3-Carbinol and Promotes Growth

**DOI:** 10.3390/foods13050696

**Published:** 2024-02-24

**Authors:** Rui Li, Zihuan Zhou, Xiaofei Zhao, Jing Li

**Affiliations:** College of Life Sciences, Northeast Agricultural University, Harbin 150030, China; liruigyh@neau.edu.cn (R.L.); ahuan_zh@163.com (Z.Z.); xiaofeizhao98@163.com (X.Z.)

**Keywords:** broccoli, methionine, tryptophan, glucosinolates, sulforaphane, indole-3-carbinol

## Abstract

Broccoli is a popular cruciferous vegetable that is well known for its abundant health-promoting biochemicals. The most important of these beneficial biochemicals are glucosinolates, including glucoraphanin and glucobrassicin. Glucoraphanin and glucobrassicin can be broken down by myrosinases into sulforaphane and indole-3-carbinol, which have been demonstrated to have potent cancer-preventive properties. Efforts to increase glucoraphanin in broccoli seedlings have long been a focus; however, increasing glucoraphanin and glucobrassicin simultaneously, as well as enhancing myrosinase activity to release more sulforaphane and indole-3-carbinol, have yet to be investigated. This study aims to investigate the impact of the combined application of tryptophan and methionine on the accumulation of sulforaphane and indole-3-carbinol, as well as their precursors. Furthermore, we also examined whether this application has any effects on seedling growth and the presence of other beneficial compounds. We found that the application of methionine and tryptophan not only increased the glucoraphanin content by 2.37 times and the glucobrassicin content by 3.01 times, but that it also caused a higher myrosinase activity, resulting in a1.99 times increase in sulforaphane and a 3.05 times increase in indole-3-carbinol. In addition, better plant growth and an increase in amino acids and flavonoids were observed in broccoli seedlings with this application. In conclusion, the simultaneous application of tryptophan and methionine to broccoli seedlings can effectively enhance their health-promoting value and growth. Our study provides a cost-effective and multi-benefit strategy for improving the health value and yield of broccoli seedlings, benefiting both consumers and farmers.

## 1. Introduction

Sprouts and microgreens, being microscale vegetables, have gained widespread acceptance among consumers due to their freshness, convenience, and health benefits, and have become increasingly popular worldwide in recent years. Sprouts refer to seedlings that are obtained from germinated seeds that are between two and seven days old, with developing cotyledons. Slightly different from sprouts, microgreens are seedlings that are between seven and twenty-one days old, with fully extended cotyledons and the first true leaves showing [1,2,3]. These small-sized vegetables are highly sought after due to their various advantages, including indoor cultivation, reduced use of pesticides and insecticides, decreased food waste production, and excellent health benefits [4].

Sprouts and microgreens of broccoli are abundant in phytonutrients, including glucosinolates, flavonoids, vitamins, and carotenoids, making them especially prized [5]. Glucosinolates are a group of amino acid-derived secondary metabolites. Based on the structure of the amino acid side chain, glucosinolates can be divided into aliphatic glucosinolates derived from methionine (Met), indole glucosinolates derived from tryptophan (Trp), and aromatic glucosinolates derived from phenylalanine [6]. The predominant glucosinolates that are present in broccoli are glucoraphanin, a type of aliphatic glucosinolate, and glucobrassicin, a type of indole glucosinolate. These can be hydrolyzed by myrosinases to produce sulforaphane (SFN) and indole-3-carbinol (I3C), respectively. Numerous studies have demonstrated that both SFN and I3C possess potent cancer-preventive activity [7,8,9,10,11].

Studies have shown that the concentration of glucoraphanin in broccoli seedlings is much higher than in mature plants. In different broccoli cultivars, it can reach 10–100 times the amount that is found in adult plants [10,12]. This makes broccoli seedlings a valuable source of functional food with anticancer properties. However, compared to mature broccoli, seedlings contain much lower quantities of glucobrassicin, the precursor of I3C, which also has anticancer properties [10,11]. Efforts to increase the amount of glucoraphanin in broccoli seedlings have long been a focus, such as utilizing different wavelengths of light, applying sulfur fertilizer, or applying CaCl_2_ [13,14,15]. However, these methods could not increase the contents of both glucoraphanin and glucobrassicin simultaneously.

Met and Trp are precursors of the anticancer compounds glucoraphanin and glucobrassicin, respectively, and therefore, they have the potential to be utilized to increase the contents of these two beneficial compounds in broccoli seedlings. Additionally, Met and Trp are also the origin of a wide variety of primary and secondary metabolites that are essential for plant growth, development, and adaptation to the environment [16,17].

The metabolic pathway of Trp has been well established in plants [17,18]. First, Trp is the main precursor of the phytohormone indole-3-acetic acid (IAA). It can be converted to IAA through the indole-3-pyruvic acid (IPYA) pathway or the indole-3-acetaldoxime (IAOx) pathway. Second, Trp is also the precursor of melatonin (N-acetyl-5-methoxytryptamine), which is considered a potential phytohormone and has been demonstrated to protect plants from various environmental stressors [19,20]. For humans, melatonin has enormous health benefits, including improved sleep, neuroprotection, tumor suppression, and anti-inflammatory action [21,22].

Met is the codon that initiates protein translation, making it a critical component of protein synthesis. In plants, Met is involved in the production of the phytohormone ethylene and polyamines, which are defensive compounds [23]. Therefore, Met plays an important role in various physiological processes.

To summarize, supplementing the supply of Trp and Met to broccoli seedlings is expected to be highly advantageous. Not only does it boost the production of the cancer-preventive compounds glucoraphanin and glucobrassicin, but it also fosters the formation of other health-promoting phytonutrients. However, the effects of external application of Met and Trp in broccoli seedlings have yet to be determined.

In this study, we found that the application of Trp raised the contents of I3C and its precursor, glucobrassicin. The application of Met increased the contents of SFN and its precursor, glucoraphanin. The addition of Met and Trp together not only caused an increase in SFN and I3C, but also enhanced the contents of their precursors glucoraphanin and glucobrassicin, as well as the myrosinase activity. The increased glucoraphanin, glucobrassicin, and myrosinase activity could potentially enhance the formation of SFC and I3C, thus improving the efficacy of these compounds in exerting their anticancer benefits. Additionally, as a precursor, Trp promotes the biosynthesis of the phytohormone IAA, thereby increasing the yield. Furthermore, the application of Trp also led to a marked increase in the content of flavonoids, which are another class of secondary metabolites with high health benefits. In short, our study provides a simple and efficient strategy that not only maximizes the accumulation of anticancer compounds, but also enhances the contents of other health-promoting chemicals and increases the yield of broccoli seedlings.

## 2. Materials and Methods

### 2.1. Plant Materials and Growth Conditions

Broccoli seeds were obtained from Zhihe Seed Company (Beijing, China). After being disinfected and vernalized for three days at 4 °C, the seeds were then cultivated on Murashige and Skoog (MS) medium in a controlled environment at 22 °C with 100 μmol photons m^−2^·s^−1^ white light and a 16 h light/8 h dark photoperiod.

### 2.2. Tryptophan (Trp) and Methionine (Met) Treatment

Seven-day-old broccoli seedlings were transferred to MS medium containing either 100 mM Trp, 100 mM Met, or both, as well as to MS medium as a control for a period of five days. Afterwards, the aerial parts of the broccoli seedlings were sampled for subsequent experiments, and the measurement indicators are shown in Figure S1.

### 2.3. Determination of Amino Acids

The samples were frozen in liquid nitrogen and then ground. The amino acids were then extracted using 20% ethanol with 1 mM HCl, and the extracts were centrifuged for 5 min at 12,000× *g* and 4 °C. The content of amino acids was determined by liquid chromatography–tandem mass spectrometry (LC-MS/MS) (API 4000, AB Sciex, Framingham, MA, USA). Amino acids in the extraction solutions were separated by a Thermo Scientific HILIC column (100 mm × 3.0 mm, 2.7 μm, Thermo Fisher Scientific, Waltham, MA, USA). Solvent A consisted of 75% acetonitrile, while solvent B consisted of 0.1 M sodium acetate. The LC gradient program was set as follows: 0 min, 10% of solvent A; 5 min, 50% of solvent A; 8 min, 50% of solvent A; and 8.1 min, 10% of solvent A; the flow rate was 0.3 mL min^−1^. The ion modes and *m*/*z* values of the precursor and product ions for each metabolite are listed in Table S1. The concentration of the amino acids was analyzed in accordance with the procedure described previously [24].

### 2.4. Determination of Indole-3-Acetic Acid (IAA) and Its Precursors

The fresh samples were collected and frozen in liquid nitrogen. After grinding, IAA and its precursors, indole-3-pyruvate (IPYA), indole-3-acetamide (IAM), and indole-3-acetonitrile (IAN), were extracted with acetonitrile and centrifuged for 5 min at 12,000× *g* and 4 °C. The extraction solutions were concentrated with nitrogen and dissolved in 400 μL of methanol. IAA and its precursors in the extract solutions were separated using an Agilent Poroshell 120 EC-C18 column (150 mm × 2.1 mm, 2.7 μm, Agilent Technologies, Santa Clara, CA, USA). Solvent A consisted of methanol with 0.1% (*v*/*v*) formic acid, while solvent B consisted of water with 0.1% (*v*/*v*) formic acid. The LC gradient program was set as follows: 0–1 min, 20% of solvent A; 1–3 min, 20–50% of solvent A; 3–9 min, 50–80% of solvent A; 9–10.5 min, 80% of solvent A; 10.5–10.6 min, 80–20% of solvent A; and 10.6–13.5 min, 20% of solvent A; the flow rate was 0.3 mL min^−1^. The ion modes and *m*/*z* values of the precursor and product ions for each metabolite are listed in Table S1. The contents were quantified through LC-MS/MS (API 4000), as outlined in [25].

### 2.5. Extraction and Analysis of Glucosinolates

Approximately 200 mg of fresh samples of broccoli seedlings were weighed, frozen immediately in liquid nitrogen, and stored at −80 °C. Glucosinolates were extracted with 5 mL of pre-cooled 80% methanol, and the extract was passed through DEAE Sephadex columns, followed by treatment with sulfatase (Sigma-Aldrich, Saint Louis, MO, USA). Extract solutions (5 μL) were then analyzed using ultra-high-performance liquid chromatography (UPLC) (1290 Infinity II, Agilent Technologies) according to the procedure previously described [26], and sinigrin (Sigma, USA) was used as an internal standard. The mobile phases were water (A) and methanol (B), and the gradient program was set as follows: 0 min, 0% solvent B; 7 min, 25% solvent B; 8.6 min, 60% solvent B; 9.2 min, 100% solvent B; 9.2–9.8 min, 100% solvent B; 10.6 min, 0% solvent B; and 10.6–13.0 min, 0% solvent B. The flow rate was 0.4 mL min^−1^, and desulfoglucosinolates were identified using a UV detector at 229 nm.

### 2.6. Determination of Melatonin and Its Precursors

About 1 g of fresh samples of broccoli seedlings were collected and ground. Melatonin and its precursors, tryptamine, serotonin, and *N*-acetylserotonin, were then extracted using methanol and quantified with LC-MS/MS (API 4000), as described in Yu et al. [27]. After centrifugation for 5 min at 8000× *g* and 4 °C, the supernatants were dried with a Termovap Sample Concentrator and dissolved in 200 μL of methanol. Melatonin and its precursors in the extraction solutions were separated using an Agilent Poroshell 120 EC-C18 column (150 mm × 2.1 mm, 2.7 μm, Agilent Technologies). Solvent A consisted of water with 0.1% (*v*/*v*) formic acid, while solvent B consisted of methanol. The LC gradient program was set as follows: 0–2 min, 20% of solvent A; 2–5 min, 20% of solvent A; and 5.1–12 min, 80% of solvent A; the flow rate was 0.3 mL min^−1^. The ion modes and *m*/*z* values of the precursor and product ions for each metabolite are listed in Table S1. The method for the calculation of melatonin and its precursors was adopted from Kang et al. [28].

### 2.7. Myrosinase Activity Assay

Frozen broccoli seedling leaves were ground and suspended in pre-cooled extraction buffer containing 50 mM PIPES (pH 7.0), 150 mM NaCl, and cOmplete Protease Inhibitor Cocktail (Roche, Basel, Switzerland). The mixture was centrifuged at 15,000× *g* at 4 °C for 10 min, and the supernatant was used for myrosinase activity determination. The activity of myrosinase was measured using the LabAssay™ Glucose Kit (FUJIFILM Wako Chemicals, Osaka, Japan) as described by Sugiyama et al. [29]. The protein concentration was measured using the Bradford assay.

### 2.8. Determination of Sulforaphane (SFN) and Indole-3-Carbinol (I3C)

Approximately 500 mg of fresh samples was collected for use. The samples were ground and suspended in 2 mL of pre-cooled 80% methanol overnight. The mixture was centrifuged at 10,000× *g* at 4 °C for 10 min, and the supernatants were dried with a nitrogen flow and then dissolved in 200 μL of methanol. The metabolites were separated by a Compass C18 (2) column (250 mm × 4.6 mm, 5 μm). Solvent A consisted of water with 0.1% (*v*/*v*) phosphoric acid, while solvent B consisted of acetonitrile. The LC gradient program was set as follows: 0 min, 80% of solvent A and 25 min, 5% of solvent A; flow rate was 1 mL min^−1^. Ion modes and *m*/*z* values of the precursor and product ions for each metabolite are listed in Table S1. The SFN and I3C contents were quantified using high-performance liquid chromatography (HPLC) (Waters 2695, Milford, MA, USA), as described previously by Zheng et al. [30].

### 2.9. Determination of Flavonoids

Samples of broccoli seedlings were ground and suspended in methanol containing 1% HCl. After centrifugation for 30 min at 11,000× *g* and 4 °C, 100 μL of the supernatant was used for determination. Flavonoids including gallic acid, protocatechuic acid, protocatechualdehyde, chlorogenic acid, caffeic acid, homoorientin, neohesperidin, hyperoside, quercetin, bergapten, kaempferol, umbelliferone, nobiletin, genistin, psoralen, asiatic acid, rhein, galangin, emodin, and chrysophanol were then detected using LC-MS/MS (API 4000) in accordance with Davuluri et al. [31]. Metabolites in the extract solution were separated using an Agilent ZORBAX Eclipse Plus C18 column (150 mm × 2.1 mm, 3.5 μm, Agilent Technologies). Solvent A consisted of water with 0.3% (*v*/*v*) formic acid, while solvent B consisted of acetonitrile with 0.3% (*v*/*v*) formic acid. The LC gradient program was set as follows: 0 min, 20% of solvent B; 1 min, 90% of solvent B; 5 min, 90% of solvent B; 5.1 min, 20% of solvent B; and 10 min, 20% of solvent B; flow rate was 0.3 mL min^−1^. Ion modes and *m*/*z* values of the precursor and product ions for each metabolite are listed in Table S1.

### 2.10. Quantitative Real-Time Polymerase Chain Reaction Analysis

Total RNA was extracted using the RNA Easy Fast Kit (Tiangen, Beijing, China). First-strand cDNA was synthesized using the ReverTra Ace qPCR RT Master Mix with a gDNA Remover Kit (Toyobo, Osaka, Japan). Quantitative real-time polymerase chain reaction (qPCR) was performed using 2× SYBR Green qPCR Mix (SparkJade, Qingdao, China) on an ABI QuantStudio™ System (Applied Biosystems, Carlsbad, CA, USA), and *BoACTIN2* was used as a reference gene. The primers used are listed in Table S2.

### 2.11. Statistical Analysis

For all experiments, three or five biological replicates and three technical replicates were conducted, and the results are presented as the mean with standard error. Significant differences were determined using Duncan’s multiple-range test (*p* < 0.05) and are denoted by different letters [32].

## 3. Results

### 3.1. Effect of Met and Trp Application on Indole-3-Acetic Acid and Melatonin Metabolism and Growth

**Figure 1:**
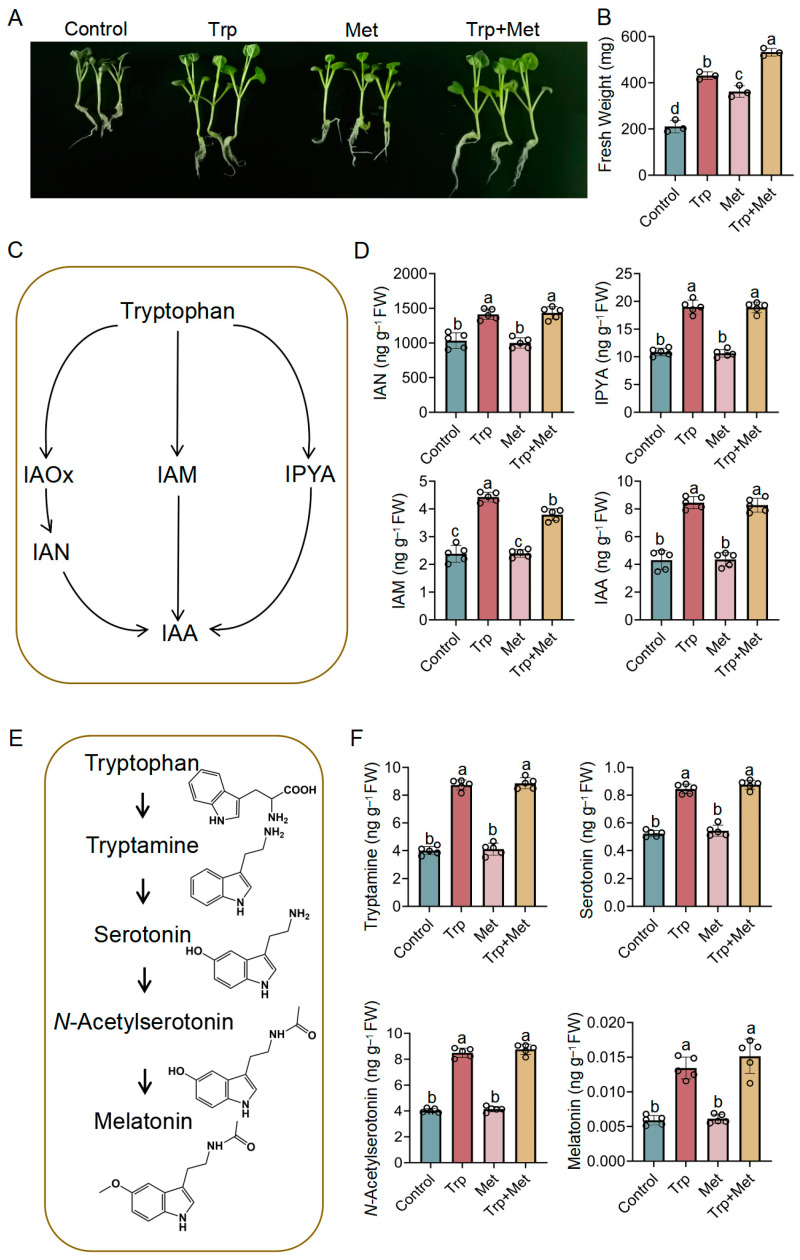
The growth of broccoli seedlings under Trp and Met treatment. (**A**) The effect of Trp and Met application on broccoli seedlings. (**B**) Fresh weight of 12-day-old broccoli seedlings. (**C**) Schematic diagram of the biosynthetic pathway of IAA. (**D**) Contents of IAN, IPYA, IAM, and IAA. (**E**) Schematic diagram of the biosynthetic pathway of melatonin. (**F**) Contents of tryptamine, serotonin, *N*-acetylserotonin, and melatonin. The lowercase letters indicate statistical significance corresponding to Duncan’s multiple-range test (*p* < 0.05). FW, fresh weight; IAA, indole-3-acetic acid; IAM, indole-3-acetamide; IAN, indole-3-acetonitrile; IPYA, indole-3-pyruvic acid.

To investigate the effect of Met and Trp on broccoli seedling growth, seven-day-old broccoli seedlings were grown on MS medium with either Trp, Met, or both. The growth of the seedlings was observed after five days. As shown in Figure 1A,B, the fresh weight of seedlings treated with Trp, Met, or both was significantly higher than that of untreated seedlings. The highest weight was 2.53 times higher compared to untreated seedlings when both Trp and Met were applied (Figure 1B). Combining Met and Trp had a cooperative effect that significantly promoted the growth of broccoli seedlings and increased the yield, which was higher than when using Trp or Met alone.

Trp is the precursor of the phytohormone IAA (Figure 1C). To investigate whether exogenous Trp can activate IAA biosynthesis, we determined the contents of IAA and intermediate products in the biosynthetic pathway. The results showed that Trp caused a considerable increase in the IAA content in broccoli seedlings (Figure 1D). We further analyzed the contents of the intermediate products IAN (indole-3-acetonitrile), IPYA, and IAM (indole-3-acetamide) in the IAA biosynthetic pathway and found that Trp treatment significantly increased the contents of IAN, IPYA, and IAM (Figure 1D), suggesting an enhancement of the entire IAA biosynthetic pathway. No significant changes in IAA content were observed after Met treatment, and there was no additive effect when Trp and Met were added together. These results indicate that Trp application activates the IAA biosynthesis pathway, thus significantly promoting the growth of broccoli seedlings.

In both plants and animals, Trp is a precursor of melatonin [33,34]. Melatonin can not only effectively treat sleep disorders in humans, it also plays an important role in plant growth and stress resistance [25]. Thus, the concentration of melatonin and the three intermediates in its biosynthetic pathway (Figure 1E) was measured. As shown in Figure 1F, the application of Trp increased the contents of melatonin and all its three precursors: tryptamine, serotonin, and *N*-acetylserotonin. Met had no significant effect on melatonin and the intermediates. There was no difference between the combination of Trp and Met and Trp alone. Even though the exogenous application of Trp caused a notable increase in the melatonin content, compared to other plants (usually ranging from a few to several thousand nanograms per gram of tissue) [35], the amount of melatonin in broccoli seedlings is still quite low. Therefore, broccoli seedlings are not an ideal source of melatonin for a daily diet. Nevertheless, melatonin was reported to be highly effective at scavenging ROS in plants under various environmental stresses [25]; thus, the augmentation of melatonin through Trp treatment could improve a plant’s adaptability to adversity.

### 3.2. Effect of Met and Trp Application on Glucosinolates and Their Degraded Products

**Figure 2:**
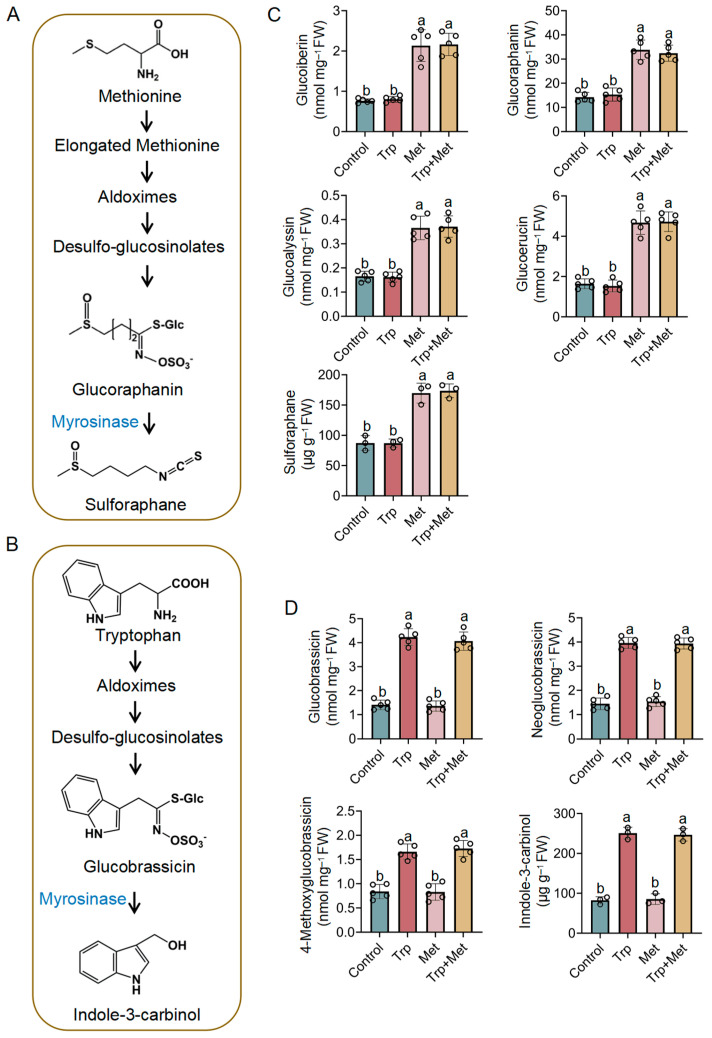
The change in glucosinolate content in broccoli seedlings under Trp and Met application. (**A**) Schematic diagram of the metabolic pathway of aliphatic glucosinolates. (**B**) Schematic diagram of the metabolic pathway of indole glucosinolates. (**C**) Contents of glucoiberin, glucoraphanin, glucoalyssin, glucoerucin, and sulforaphane. (**D**) Contents of glucobrassicin, neoglucobrassicin, 4-methoxyglucobrassicin, and indole-3-carbinol. The lowercase letters indicate statistical significance corresponding to Duncan’s multiple-range test (*p* < 0.05). FW, fresh weight.

Since Met and Trp are precursors of aliphatic and indole glucosinolates, respectively [36] (Figure 2A,B), in order to determine whether exogenous Met and Trp can promote the biosynthesis of aliphatic and indole glucosinolates, the contents of aliphatic and indole glucosinolates in broccoli seedlings that were treated with Trp, Met, or both was determined. Compared to the control, the contents of aliphatic glucosinolates (glucoiberin, glucoraphanin, glucoalyssin, and glucoerucin) in broccoli seedlings that were treated with Met significantly increased (Figure 2C), with the content of glucoraphanin, a precursor of the anticancer compound SFN, increasing to 2.37 times that of the untreated group. The content of SFN, a degradation product of glucoraphanin with various anticancer abilities, was also detected. The application of Met significantly promoted the production of SFN, with a nearly 2-fold increase in content. Trp treatment had no discernible impact on the four aliphatic glucosinolates and SFN. In conclusion, Met treatment simultaneously enhanced the contents of SFN and its precursor glucoraphanin, greatly improving the potential anticancer activity of broccoli seedlings.

The contents of indole glucosinolates (glucobrassicin, neoglucobrassicin, and 4-methoxyglucobrassicin) and I3C were also measured. As shown in Figure 2D, the contents of all four indole glucosinolates significantly increased after treatment with Trp. As the precursor of the anticancer active substance I3C, the content of glucobrassicin increased to 3.01 times that of the untreated group under Trp treatment. Additionally, the content of I3C under Trp treatment was 3.05 times higher than that of the control group. Met had no effect on the changes in the contents of any of the indole glucosinolates or I3C. It can be concluded that the application of Trp greatly increased the contents of glucobrassicin and I3C, which has the potential to enhance its anticancer ability.

### 3.3. Effect of Met and Trp Application on Myrosinase Activity

**Figure 3:**
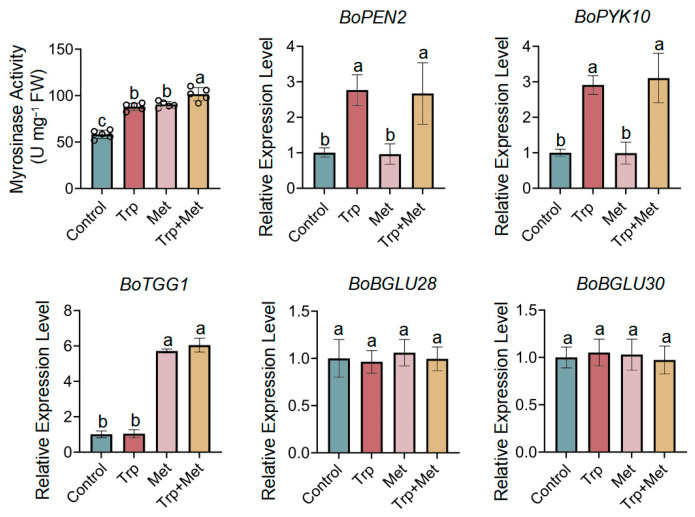
Effects of Trp and Met application on the glucosinolate degradation in broccoli seedlings. Myrosinase activity and the expression level of genes encoding myrosinases are shown. The lowercase letters indicate statistical significance corresponding to Duncan’s multiple-range test (*p* < 0.05). FW, fresh weight.

Intact glucosinolates are generally believed to have no biological activity [34], and they need to be degraded by myrosinases before releasing anticancer compounds. Therefore, the activity of myrosinase greatly affects the potential anticancer properties of broccoli seedlings. We therefore measured the myrosinase activity in broccoli seedlings after treatments with Trp, Met, or both (Figure 3). All three treatments effectively promoted the activity of myrosinase, with the combination of Trp and Met showing the greatest effect.

PEN2 and PYK10 were found to be the main myrosinases that catalyze indole glucosinolates [37], while TGG1, BGLU28, and BGLU30 were identified as the myrosinases that catalyze aliphatic glucosinolates [29,38,39]. To further investigate which myrosinases are responsible for the increase in activity that is mediated by Met and Trp, the expressions of *BoPEN2*, *BoPYK10*, *BoTGG1*, *BoBGLU28*, and *BoBGLU30* were determined in broccoli seedlings treated with Trp, Met, or both. Figure 3 shows that the expressions of *BoPEN2* and *BoPYK10* were significantly induced by Trp and not affected by Met, while *BoTGG1* expression was markedly enhanced by Met and not affected by Trp. There was no significant change in the gene expressions of *BoBGLU28* and *BoBGLU30* under all different treatments, suggesting that BoBGLU28 and BoBGLU30 are not involved in the increase in myrosinase activity that is mediated by Trp or Met. These results indicate that Trp can promote the degradation of indole glucosinolates by activating *BoPEN2* and *BoPYK10*, while Met can stimulate the degradation of aliphatic glucosinolates by inducing *BoTGG1*. Furthermore, the increase in myrosinase activity may be caused by the increased availability of its substrates, which should greatly improve the anticancer activity of broccoli seedlings.

### 3.4. Effect of Met and Trp Application on the Amino Acid Content

**Figure 4:**
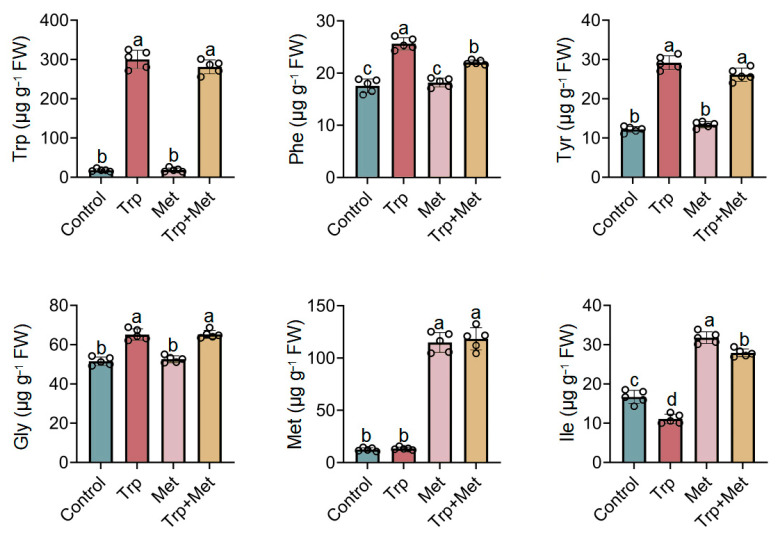
Effects of Trp and Met on the contents of amino acids in broccoli seedlings. Contents of Trp, Phe, Tyr, Gly, Met, and Ile are shown. The lowercase letters indicate statistical significance corresponding to Duncan’s multiple-range test (*p* < 0.05). FW, fresh weight; Gly, glycine; Ile, isoleucine; Met, methionine; Phe, phenylalanine; Trp, tryptophan; Tyr, tyrosine.

Amino acids are involved in the formation of proteins, and their intake plays an important role in human growth and development [40]. Therefore, the contents of twenty amino acids in broccoli seedlings were determined. As shown in Figure 4, treatment with Trp resulted in an increase in Trp, Phe, tyrosine (Tyr), and glycine (Gly) contents, and a slight decrease in isoleucine (Ile) content. On the other hand, treatment with Met resulted in an increase in the contents of Met and Ile. When Trp and Met were applied together, a comparable effect on Trp, Phe, Tyr, Gly, Met, and Ile contents was observed, similar to when either was used alone. Regarding the other fourteen amino acids, whether treated with Trp or Met alone or together, no significant changes were observed in their contents (Figure S2). In conclusion, Trp and Met had distinct impacts on the contents of amino acids, and the combined application of Trp and Met could significantly enhance the levels of several important amino acids, thereby increasing the health value of broccoli seedlings.

### 3.5. Effect of Met and Trp Application on Flavonoid Content

To determine the effect of Trp and Met on flavonoid accumulation, we measured the content of flavonoids after treatments. Table 1 displays the contents of 12 detectable flavonoids, namely, gallic acid, protocatechuic acid, protocatechualdehyde, chlorogenic acid, caffeic acid, homoorientin, neohesperidin, hyperoside, quercetin, bergapten, kaempferol, and umbelliferone. The other eight flavonoids, nobiletin, genistin, psoralen, asiatic acid, rhein, galangin, emodin, and chrysophanol, had either very low content or were undetectable. As shown in Table 1, after Trp treatment, the content of all 12 detected flavonoids significantly increased, with chlorogenic acid, homoorientin, hyperoside, quercetin, bergapten, and kaempferol increasing by more than two times. Met treatment had no significant effect on any flavonoids, and there were no differences between the combination of Trp and Met and Trp alone. These findings suggest that Trp application effectively promotes the accumulation of flavonoids in broccoli seedlings, thereby further enhancing their health benefits.

## 4. Discussion

### 4.1. Application of Trp and Met Is Beneficial in Augmenting the Content of Anticancer Compounds

Trp and Met are precursors of many beneficial metabolites, with glucosinolates being the most prominent among them [41]. SFN and I3C, which are degradation products derived from aliphatic glucosinolate glucoraphanin and indole glucosinolate glucobrassicin [35], have been demonstrated to have potent anticancer properties, and the mechanisms behind them are well understood [30,42]. Broccoli seedlings are especially rich in glucoraphanin, but slightly rich in glucobrassicin, showing great potential to be developed into an anticancer functional food. Therefore, developing effective strategies to further increase the production of glucoraphanin and glucobrassicin has far-reaching significance.

Increasing the content of glucoraphanin has long been a focus [43]. Studies have revealed that certain environmental stressors, such as salinity, drought, and hormones like MeJA and ABA, can stimulate the production of glucoraphanin [25,44,45]. However, these stressors and hormones also inhibit plant growth and, consequently, decrease yield. Trp and Met serve as precursors of the glucosinolate metabolism. In this study, applying Trp and Met to broccoli seedlings directly promotes the entire glucosinolate metabolic pathway. At the same time, the growth of the seedlings is promoted, and their fresh weight is significantly increased.

When broccoli is consumed, cell damage causes the contact of glucosinolates and myrosinases, which are spatially isolated, resulting in the degradation of glucosinolates and the release of cancer-preventive SFN and I3C [46]. The effectiveness of myrosinase can be reduced or eliminated when broccoli is cooked, depending on the cooking method and time [47]. In contrast, broccoli sprouts usually do not require cooking and are ready to eat; therefore, the myrosinase activity is crucial for their anticancer potential. Our study showed that the application of Trp and Met not only increased the contents of the anticancer compounds SFN and I3C, as well as their precursors glucoraphanin and glucobrassicin, but also enhanced myrosinase activity. Furthermore, in the treated seedlings, the gene expressions of myrosinases, which catalyze the degradation of aliphatic glucosinolates, and myrosinases, which catalyze the degradation of indole glucosinolates, both increased, indicating that both glucoraphanin and glucobrassicin are more likely to be degraded to release SFN and I3C.

In summary, the exogenous application of Trp and Met is beneficial for improving the anticancer properties of broccoli seedlings in multiple ways: (ⅰ) Simultaneously increasing the contents of two anticancer compounds, SFN and I3C. Due to the synergistic effect between SFN and I3C [48], the increase in their contents will greatly benefit the improvement of broccoli seedlings’ anticancer efficacy. (ⅱ) Boosting the contents of glucoraphanin and glucobrassicin, which are precursors to SFN and I3C. (ⅲ) Enhancing myrosinase activity, enabling glucoraphanin and glucobrassicin to be effectively degraded to release SFN and I3C.

### 4.2. Application of Trp and Met Promotes Accumulation of Other Beneficial Phytochemicals in Broccoli Seedlings

The application of Trp and Met not only increases the content of anticancer glucosinolates, but also enhances the accumulation of other beneficial phytochemicals, thereby further improving the health-promoting value of broccoli seedlings.

Flavonoids are derived from phenylalanine (Phe), which shares a common precursor, chorismate, with Trp. Increasing the supply of Trp will reduce the consumption of chorismate, thus promoting the biosynthesis of Phe and facilitating the production of flavonoids [49]. Our study showed that Trp can significantly increase the content of flavonoids. Flavonoids have been identified as therapeutics due to their biological activities, such as anti-inflammatory, neuroprotective, and cardioprotective effects [50,51]. Therefore, augmenting the flavonoid content in broccoli would further enhance its health value.

As materials in protein biosynthesis, amino acids are essential nutrients that are required for human growth and development [52]. The application of Trp and Met significantly increases the contents of several amino acids, consequently enhancing the nutritional value of broccoli seedlings. It is noteworthy that, after being applied exogenously, apart from being converted into beneficial compounds such as glucosinolates, the excess Trp accumulates in the plant. Trp is an amino acid that cannot be synthesized in humans and animals and therefore must be obtained from plants. Generally, the Trp content is quite low in many crops and vegetables, which is inadequate to meet the demands of humans and animals. In humans, Trp is the precursor of various proteins and metabolites, including tryptamine, serotonin, melatonin, and quinolinic acid [5,35]. A deficiency of Trp can lead to a range of diseases, including sleep disorders, mental disorders, and pellagra, all of which can have serious impacts on human health [53]. Therefore, the enhanced accumulation of Trp in broccoli seedlings is beneficial for providing this essential amino acid in a safe manner.

### 4.3. Application of Trp and Met Improves Yield and Potential Stress Tolerance of Broccoli Seedlings

IAA, the foremost natural auxin in plants, has been discovered to be critical for plant growth and development. In the absence of IAA, plant growth is significantly hindered; thus, Trp is necessary to maintain the growth and development of plants [17]. The IAA content of broccoli under Trp application was much higher than that of the control, thereby increasing the growth of broccoli, which is beneficial for improving its yield.

Melatonin is known to be involved in the regulation of plants’ circadian rhythm, seed germination, biomass increase, and response to biotic and abiotic stresses [19,20]. Therefore, the effect of Trp treatment on the melatonin content in broccoli is of great significance in improving resistance to stress. Although hydroponically grown and home-grown microgreens rarely encounter drought, salinity, low temperatures, etc., they may be prone to diseases and pests. Thus, increasing glucosinolates and downstream related products in broccoli seedlings is beneficial for improving resistance to biotic stresses. As a result, our research not only improves the health-promoting value of broccoli, but also enhances its potential stress resistance.

## 5. Conclusions

**Figure 5:**
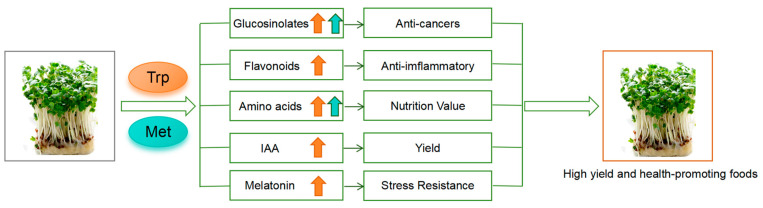
A model for the effects of Trp and Met supplementation on the growth and health value of broccoli. The orange arrow represents the increase in the content of metabolites caused by the application of Trp. The cyan arrow represents the increase in the content of metabolites caused by the application of Met.

In conclusion, the application of Trp and Met has multiple benefits for broccoli seedlings. It increases the production of anticancer substances, resulting from the simultaneous increase in glucoraphanin and glucobrassicin, as well as the enhancement of myrosinase activity. Additionally, the increased production of various amino acids, flavonoids, IAA, and melatonin is beneficial for improving the yield and enhancing the health-promoting values of broccoli (Figure 5). Our study provides a cost-effective and safe strategy for enhancing the health value of broccoli.

## Figures and Tables

**Table 1:** Effects of Trp, Met, and Trp plus Met application on the content of flavonoids in broccoli.

Flavonoid Content (ng g^−1^ FW)	Control	Trp Treatment	Met Treatment	Trp + Met Treatment
Gallic acid	12.03 ± 1.16	18..69 ± 1.33 **	11.79 ± 1.28	17.40 ± 1.65 **
Protocatechuic acid	12.00 ± 1.17	18.12 ± 1.51 **	11.82 ± 1.28	17.98 ± 1.80 **
Protocatechualdehyde	7.18 ± 0.48	12.10 ± 1.04 **	7.09 ± 0.37	11.96 ± 0.72 **
Chlorogenic acid	75.89 ± 8.08	168.07 ± 36.33 **	79.13 ± 9.26	159.62 ± 25.93 **
Caffeic acid	24.80 ± 2.51	45.83 ± 6.40 **	24.97 ± 2.95	47.77 ± 4.20 **
Homoorientin	5.13 ± 0.65	16.59 ± 2.61 **	4.99 ± 0.36	16.37 ± 0.90 **
Neohesperidin	5.91 ± 0.85	11.41 ± 0.61 **	5.98 ± 1.00	11.65 ± 0.66 **
Hyperoside	2.89 ± 0.54	6.45 ± 1.05 **	2.83 ± 0.34	6.39 ± 0.79 **
Quercetin	3.76 ± 0.48	8.39 ± 0.77 **	3.96 ± 0.58	8.44 ± 0.75 **
Bergapten	300.66 ± 34.30	975.72 ± 209.55 **	316.92 ± 41.33	899.63 ± 150.12 **
Kaempferol	11.84 ± 0.95	27.50 ± 2.60 **	11.59 ± 0.94	26.29 ± 3.50 **
Umbelliferone	5.32 ± 0.48	8.71 ± 0.64 **	5.15 ± 0.24	9.14 ± 1.02 **

Note: ** represents significant differences compared with control at *p* < 0.01 (Student’s *t*-test).

## Data Availability

The original contributions presented in the study are included in the article and Supplementary Material, further inquiries can be directed to the corresponding authors.

## Supplementary Materials

The following supporting information can be downloaded at https://www.mdpi.com/article/10.3390/foods13050696/s1, Figure S1: Study design of the experiment; Figure S2: Effect of Trp, Met, and Trp plus Met application on the content of amino acids in broccoli; Table S1: Ion modes and *m/z* values of the precursor and product ions for each metabolite analyzed by liquid chromatography–tandem mass spectrometry (LC–MS/MS); Table S2: Primers used in this study for quantitative real-time polymerase chain reaction analysis.
