# Supplementary material for: Application of Tryptophan and Methionine in Broccoli Seedlings Enhances Formation of Anticancer Compounds Sulforaphane and Indole-3-Carbinol and Promotes Growth
Source: Foods. 2024 Feb 24;13(5):696. doi: 10.3390/foods13050696 (PMC10930459; doi:10.3390/foods13050696)
Supplement: Supplementary file 1 [file foods-13-00696-s001.zip › foods-2869017-supplementary.pdf]

Supporting Information

Application of Tryptophan and Methionine in Broccoli Seedlings Enhances Formation of Anticancer Compounds Sulforaphane and Indole-3-Carbinol and Promotes Growth

Rui Li, Zihuan Zhou, Xiaofei Zhao and Jing Li *

College of Life Sciences, Northeast Agricultural University, Harbin 150030, China

* Correspondence: lijing@neau.edu.cn; Tel.: +86-451-87132018

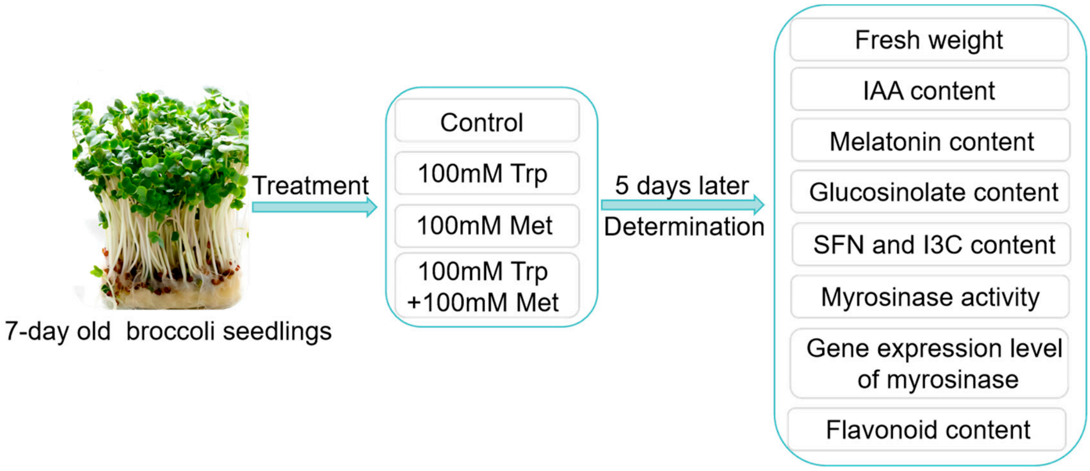

Figure S1. Study design of the experiment. IAA, indole-3-acetic acid; I3C, indole-3-carbinol; SFN, sulforaphane

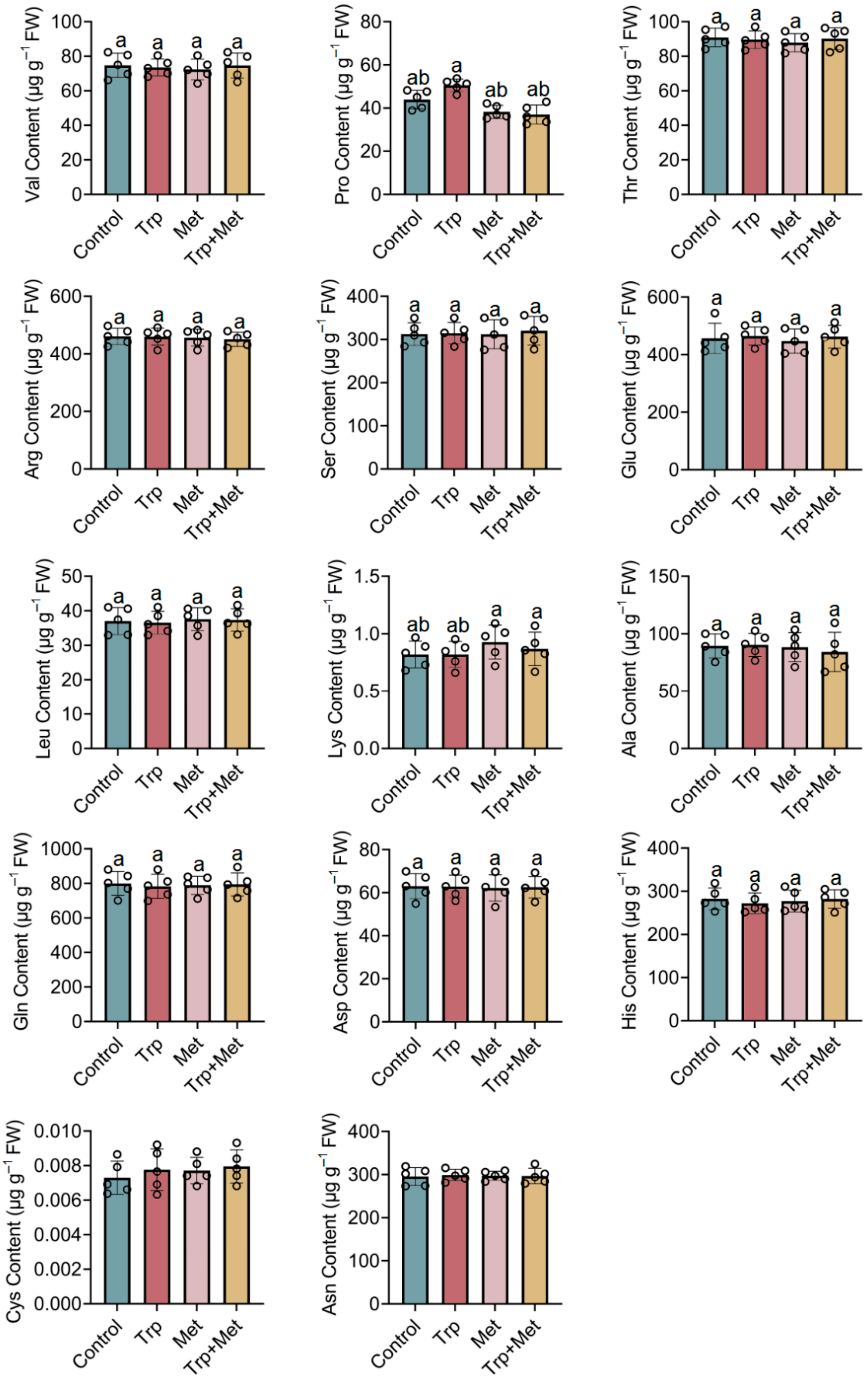

Figure S2. Effect of Trp, Met and Trp plus Met application on the content of amino acids in broccoli. The lowercase letters indicate statistical significance corresponding to Duncan's multiple-range test ($p < 0.05$). Ala, alanine; Arg, arginine; Asn, asparagine; Asp, aspartate; Cys, Cysteine; Gln, glutamine; Glu, glutamate; His, histidine; Leu, leucine; Lys, lysine; Pro, proline; Ser, serine; Thr, threonine; Val, valine

Table S1. Ion modes and *m*/*z* values of the precursor and product ions for each metabolite analyzed on a liquid chromatography–tandem mass spectrometry (LC–MS/MS).

Metabolite name	Ion mode	Precursor *m*/*z*	Product *m*/*z*
Indole-3-acetonitrile	Positive	157.1	130
Indole-3-pyruvic acid	Negative	202	115
Indole-3-acetamide	Positive	175	158
Indole-3-acetic acid	Positive	176.1	130.1
Tryptamine	Positive	161	116.9
Serotonin	Positive	177	114.9
N-Acetylserotonin	Positive	219.1	160
Melatonin	Positive	233.1	159.1
Sulforaphane	Positive	178	114
Indole-3-carbinol	Positive	170.1	130.1
Tryptophan	Positive	205.1	188
Phenylalanine	Positive	166.1	120
Tyrosine	Positive	182	165
Glycine	Positive	76	48.3
Methionine	Positive	150	133
Isoleucine	Positive	132	86.1
Valine	Positive	118	72.1
Proline	Positive	116	70.3
Threonine	Positive	120	74.1
Arginine	Positive	175.1	70.3
Serine	Positive	106	88
Glutamate	Positive	148.1	130
Leucine	Positive	132	86.1
Lysine	Positive	147	130
Alanine	Positive	90	45.3
Glutamine	Positive	147	130
Aspartate	Positive	134	88
Histidine	Positive	156	110
Cysteine	Positive	241.1	152
Asparagine	Positive	133	87
Gallic acid	Negative	169	125
Protocatechuic acid	Negative	153	109
Protocatechualdehyde	Negative	137	108
Chlorogenic acid	Negative	353.1	191
Caffeic acid	Negative	179	135
Homoorientin	Negative	447	357
Neohesperidin	Negative	609.2	301
Hyperoside	Negative	463.2	299.8
Quercetin	Negative	301	151
Bergapten	Negative	217	202
Kaempferol	Negative	593.2	285

Table S1 (continued).

Metabolite name	Ion mode	Precursor *m/z*	Product *m/z*
Umbelliferone	Negative	161	133

Table S2. Primers used in this study for quantitative real-time polymerase chain reaction analysis.

Gene	Primer Name	Primer Sequence
**Primers for gene expression analysis of glucosinolate degradation pathway**		
BoPEN2	BoPEN2-qPCR-Fw	ATGGGTGTACAGTGTTGCGG
	BoPEN2-qPCR-Rv	GCCTCGTATCCCGACATTCC
BoPYK10	BoPYK10-qPCR-Fw	TCGTCGGCCTCAACTACTACA
	BoPYK10-qPCR-Rv	GCCTCTCGCATAAACGGGAA
BoTGG1	BoTGG1-qPCR-Fw	GCTATGCTATCGGAACAG
	BoTGG1-qPCR-Rv	GTAATCATCACAGGTCCAATC
BoBGLU28	BoBGLU28-qPCR-Fw	ACGTTGCTCGAAATGGGTGA
	BoBGLU28-qPCR-Rv	AAGGCTCAAACCACAACGGT
BoBGLU30	BoBGLU30-qPCR-Fw	TGGGAACATGGGTACACTGC
	BoBGLU30-qPCR-Rv	ACGAGCAACGATTCTCCTCC
BoACTIN2	BoACTIN2-qPCR-Fw	TGTGACGTGGATATCAGGAAGGAC
	BoACTIN2-qPCR-Rv	GAACCACCGATCCAGACACTGTACT
